# Cardiovascular risk prediction in India: Comparison of the original and recalibrated Framingham prognostic models in urban populations.

**DOI:** 10.12688/wellcomeopenres.15137.2

**Published:** 2019-12-02

**Authors:** Priti Gupta, David Prieto-Merino, Vamadevan S. Ajay, Kalpana Singh, Ambuj Roy, Anand Krishnan, K.M. Venkat Narayan, Mohammed K. Ali, Nikhil Tandon, Dorairaj Prabhakaran, Pablo Perel

**Affiliations:** 1 Centre for Chronic Disease Control, New Delhi, 110016, India; 2 Centre for Global Chronic Conditions, London School of Hygiene & Tropical Medicine, London, UK; 3 Applied Statistical Methods in Medical Research Group, Universidad Catolica San Antonio de Murcia (UCAM), Murcia, Spain; 4 All India Institute of Medical Sciences, New Delhi, 110029, India; 5 Rollins School of Public Health, Atlanta, USA; 6 Public Health Foundation of India, Gurgaon, Haryana, India

**Keywords:** CVD risk score prognostic model, the re-calibrated Framingham risk score

## Abstract

**Introduction: **Cardiovascular diseases (CVDs) are the leading cause of death in India. The CVD risk approach is a cost-effective way to identify those at high risk, especially in a low resource setting. As there is no validated prognostic model for an Indian urban population, we have re-calibrated the original Framingham model using data from two urban Indian studies.

**Methods: **We have estimated three risk score equations using three different models. The first model was based on Framingham original model; the second and third are the recalibrated models using risk factor prevalence from CARRS (Centre for cArdiometabolic Risk Reduction in South-Asia) and ICMR (Indian Council of Medical Research) studies, and estimated survival from WHO 2012 data for India. We applied these three risk scores to the CARRS and ICMR participants and estimated the proportion of those at high-risk (>30% 10 years CVD risk) who would be eligible to receive preventive treatment such as statins.

**Results: **In the CARRS study, the proportion of men with 10 years CVD risk > 30% (and therefore eligible for statin treatment) was 13.3%, 21%, and 13.6% using Framingham, CARRS and ICMR risk models, respectively. The corresponding proportions of women were 3.5%, 16.4%, and 11.6%. In the ICMR study the corresponding proportions of men were 16.3%, 24.2%, and 16.5% and for women, these were 5.6%, 20.5%, and 15.3%.

**Conclusion: **Although the recalibrated model based on local population can improve the validity of CVD risk scores our study exemplifies the variation between recalibrated models using different data from the same country. Considering the growing burden of cardiovascular diseases in India, and the impact that the risk approach has on influencing cardiovascular prevention treatment, such as statins, it is essential to develop high quality and well powered local cohorts (with outcome data) to develop local prognostic models.

## Introduction

Currently, cardiovascular diseases (CVDs) account for two-thirds of the total non-communicable disease (NCD) burden in India ^1^. According to the 2016 Global Burden of Disease study, ischemic heart disease was the leading cause of the Disability-adjusted life years (DALYs), measured to be 3062 per 100,000 population in India ^2^. Also, the all-age death rate increased significantly between 1990 and 2016 for ischaemic heart disease (percentage change 54·5%), and CVDs are the leading cause of death in most parts of India ^2, 3^. Age adjusted prevalence of CVDs have also increased in India ^4, 5^. Indians are affected by CVDs at a younger age compared to their European counterparts, with more than 50% CVDs deaths occurring before the age of 70 ^6– 8^. The World Health Organization (WHO) had estimated that, due to the burden of CVDs, India had lost 237 billion dollars over ten years (2005–2015) ^9^.

CVDs risk approach is a cost-effective means to identify those at high risk so that immediate short and long-term preventive steps can be followed to mitigate the risk ^10^. Risk stratification approach has been primarily found to be cost-effective in resource-poor settings ^11^.

Although risk factor effect can be similar across populations, the estimated cardiovascular disease risk from risk models differs substantially across populations. This is mainly because of the different “baseline incidence of the risk model outcome” and prevalence of the different risk factors across populations. Also, a meta-analysis based on 17 population-based cohorts worldwide has shown that ethnicity modifies the association between risk factors and cardiovascular disease ^12^. Another study from the United Kingdom has shown the CVD risk prediction model to be inaccurate in the South Asian group as compared to white Europeans ^13^. Studies have also proved that the Framingham risk prediction model underestimates the CVD risk in Asian Indians and socioeconomically deprived individuals ^14, 15^.

The Framingham risk equation is a well-established and widely used method to measure cardiovascular disease (CVD) risk, but was developed with a white US-based population several decades ago and so, there is a need to re-calibrate it when applying it in other populations. Recalibrating a risk equation to a new population involves estimating the average values of the risk factors and the average risk of CVD. These values are used as the reference values in the risk model equations. To the best of our knowledge, only one study has recalibrated the Framingham risk equation in India, and this was for a rural population ^16^. As there is no validated prognostic model for an Indian urban population, we have re-calibrated the original Framingham model. In this paper, we report the Framingham model recalibration to an Indian urban population using data from two studies: CARRS (Centre for cArdiometabolic Risk Reduction in South-Asia), and ICMR (Indian Council of Medical Research). We compare the 10-year predictions of CVD fatal and non-fatal events produced by the original Framingham model and the recalibrated models and describe the potential impact of the recalibration on the proportion of the population eligible for treatment as recommended by current WHO guidelines.

## Methods

### Data sources

We used data from two studies:

a) The CARRS Cohort study was a population-based sample of urban adults in Chennai, New Delhi and Karachi established to assess the prevalence and incidence of cardio-metabolic diseases and their risk factors. Its details have been published previously ^17^, in brief, participants were selected in each city using multi-stage cluster random sampling with the Kish method ^18^ to select only one man and one woman aged 20+ from each randomly selected household. Here we used baseline data from the cross-sectional survey conducted between October 2010, and December 2011 with mortality follow up through June 2014.
b) The ICMR study was a cross-sectional survey conducted to estimate CVD risk factor prevalence in the National Capital Region of India (Delhi and Ballabgarh) in 2010–2012 ^19^. Multi-stage cluster random sampling was used for the primary sampling unit (household) selection. Data were collected on sociodemographic characteristics, CVD risk factors, treatment status, and measurements of height, weight, hip and waist circumference, and blood pressure. Fasting blood glucose (FBG) and lipids measurements were done using fasting venous blood. Here we have included data only from the urban area of Delhi.

### Risk calculation

Below we described the different steps we conducted to recalibrate the Framingham score

**Step 1: Prognostic index or linear predictor calculation (i.e. Xi):** We first calculated each individual Framingham “score” for CVD events in the next 10 years. This is a weighted sum of the individual’s characteristics using the Framingham weights (see Table 1).

**Table 1.** Coefficients from simplified Framingham model ^22^.

	MEN	WOMEN
Log of Age	3.11296	2.72107
Log of Body Mass Index	0.79277	0.51125
Log of SBP * if not treated	1.85508	2.81291
Log of SBP if treated	1.92672	2.88267
Smoking (0=No / 1=Yes)	0.70953	0.61868
Diabetes (0=No / 1=Yes)	0.53160	0.77763

* SBP: systolic blood pressure.

**Step 2: Reference individual survival calculation (S _0_):** We then obtained the 2012 yearly mortality rates from CVD causes for India from WHO (0.003489 for men and 0.002646 for women). We assumed that the ratio of non-fatal to fatal events was 2:1 so the yearly rates of total fatal and non-fatal events was estimated as 3*0.003489 = 0.010467 for men and 3*0.002646 = 0.007938 for women. We assumed a constant ratio, and therefore the probabilities of not having events in 10-years were (1 – 0.010467) ^10^ = 0.90013 for men and (1 – 0.007938) ^10^ = 0.923396 for women.

**Step 3: Reference individual score calculation (X _0_):** We then calculated three risks for each individual considering different values of scores and survivals: M1) using Framingham’s reference score and survival, M2) using a reference score ( *X*_0_) derived from CARRS and estimated survival ( *S*_0_) derived from WHO 2012 data for India (see above), and M3) using reference score ( *X*_0_) derived from ICMR and the estimated survival ( *S*_0_) derived from WHO 2012 data for India. We estimated the score of the “average individual” in the population by multiplying the averages of the variables (in the log scale for the continuous variables) by the original Framingham coefficients and adding the values for all risk factors.

**Step 4: Estimation of risks with different models:** We calculated the risks for everyone in the CARRS and the ICMR datasets with three models (M1, M2, M3) using different combinations of reference score ( *X*_0_) and survival probabilities ( *S*_0_).

### Comparison of risk and treatment

With each of the three risk calculations (M1. M2 and M3), we have stratified individuals in three different risk categories (<10%, 10–30%, and >30) which are commonly used for treatment recommendations for antihypertensive and statins. To see how recalibration with one or another data set affects the proportion of individuals treated, we have compared the proportion of individuals in the third risk category (>30%) between the different models. We reported the study following the TRIPOD statement ^20^. A completed TRIPOD statement is available from OSF ^21^. We used statistical package R, version 3.5.1 (2018-07-02)[1] for all our analysis

## Results

### Population characteristics

The CARRS study had data from 16,287 participants, but only 11,407 of those had the data needed to calculate the Framingham risk score (5,151 men and 6,256 women). The ICMR study had 3,075 individuals, but only 2,401 had all the data needed to calculate the score (1,089 men and 1,312 women).

In Table 2 we show the summaries statistics for each of the variables used in the Framingham score calculated in all the individuals that provided data for each variable separately.

**Table 2.** Descriptive of risk factors in the two data sets *: mean (standard deviation).

	MEN			WOMEN		
Data	CARRS	ICMR	All	CARRS	ICMR	All
Age *	42.9 (13.7)	47.2 (13.0)	43.6 (13.7)	41.1 (12.5)	45.2 (13.0)	41.8 (12.7)
BMI *	24.2 (4.6)	24.7 (6.1)	24.3 (4.9)	26.5 (5.5)	26.5 (5.5)	26.5 (5.5)
SBP * untreated	124.9 (17.7)	129.9 (19.4)	125.7 (18.0)	116.5 (17.9)	122.3 (19.6)	117.5 (18.4)
SBP * treated	139.7 (21.1)	143.9 (23.8)	140.7 (21.8)	136.5 (23.8)	143.6 (24.5)	138.1 (24.1)
Treatment	8.6%	13.8%	9.4%	13.6%	19.0%	14.5%
Smokers	27.1%	28.8%	27.3%	1.6%	3.3%	1.9%
Diabetics	26.0%	12.5%	23.5%	25.5%	10.7%	22.6%

BMI: body mass index, SBP: systolic blood pressure ***NOTE: We have removed patients with prevalent CVD from the CARRS and ICMR datasets***

In Table 3 below we report the reference scores using the Framingham, CARRS and ICMR populations, for this we have used the means of the log of the variables (which is not the same as the log of the mean). For example, for age, we first calculated a new variable "log(age)" for every single individual. Then we calculated the mean of this mean Mean[log(age)] =3.72033. Table 4 shows an example of the calculations using the CARRS population means.

**Table 3.** Reference scores using Framingham, CARRS and ICMR populations and reference survival times from Framingham and WHO.

Data	Male	Female
**Average scores ( *X*_0_)**		
Framingham	23.93880	26.01450
CARRS	23.36817	25.31639
ICMR	23.76358	25.66680
**Reference Survival ( *S*_0_)**		
Framingham	0.88431	0.94833
WHO 2012	0.90013	0.92340

CARRS: Centre for cArdiometabolic Risk Reduction in South-Asia; ICMR: Indian Council of Medical Research; WHO: World health organization

**Table 4.** Example of reference score calculations using means from CARRS population.

	MEN	WOMEN
	Coefficient * mean	Coefficient * Mean
Log of Age	3.11296*3.72033	2.72107*3.67602
Log of Body Mass Index	0.79277*3.17187	0.51125*3.25623
Log of SBP if untreated	1.85508*4.81871*0.8975	2.81291*4.74785*0.8553
Log of SBP if treated	1.92672*4.92633*0.1025	2.88267*4.90344*0.1447
Smoking	0.70953*0.26817	0.61868*0.01607
Diabetes	0.53160*0.27187	0.77763*0.26391
**Sum**	**SM = 23.42494**	**SW = 25.35218**

**Figure 1.**
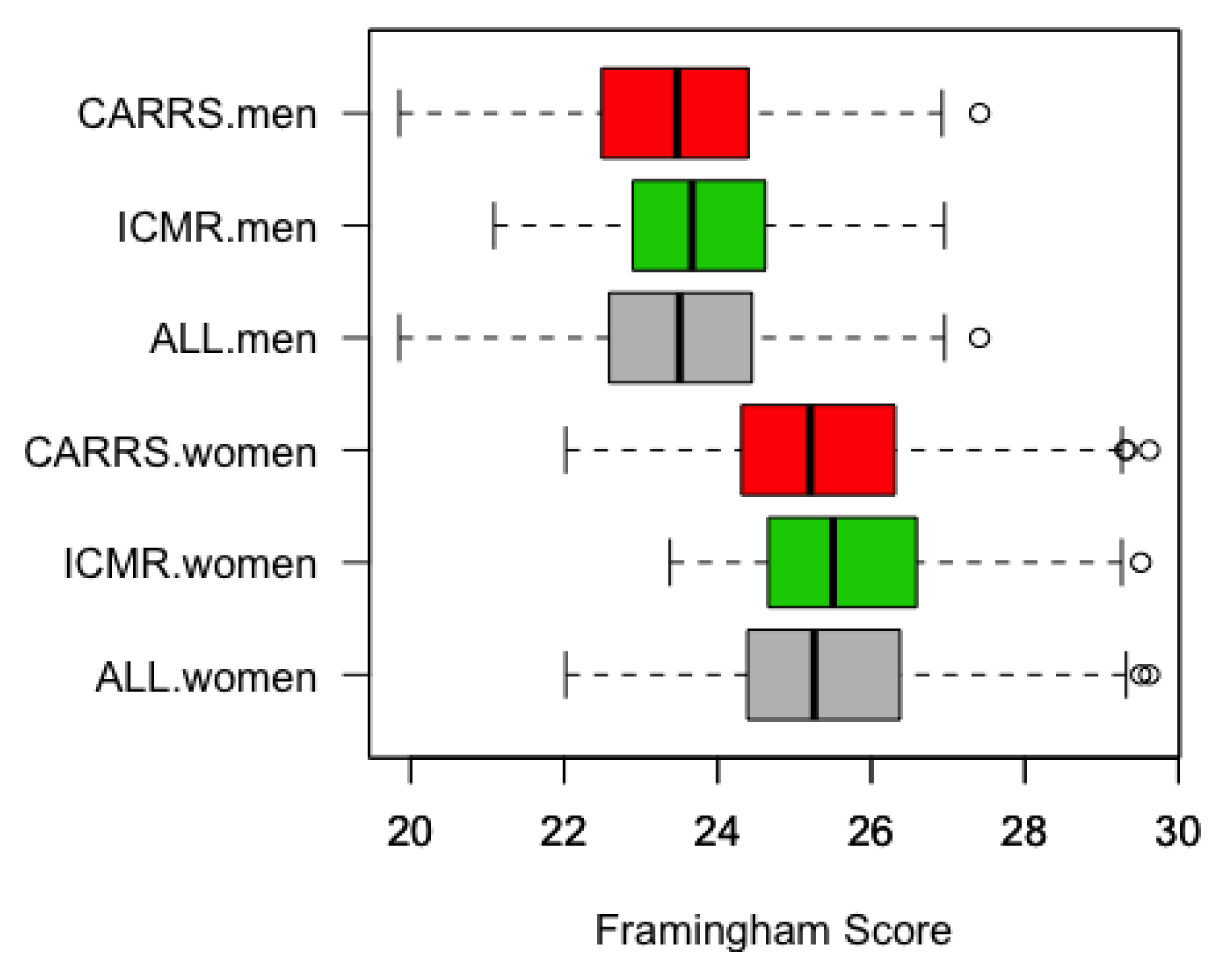
Box-plots of the distribution of the Framingham scores by sex and study. CARRS: Centre for cArdiometabolic Risk Reduction in South-Asia; ICMR: Indian Council of Medical Research.

Figure 1 shows the distribution of the recalibrated Framingham scores by sample and sex. Women have on average higher scores than men, and the Framingham ICMR recalibrated score has slightly higher means than the Framingham CARRS recalibrated in each sex.

Finally, in Table 5 we summarize the risk of the participants in both, CARRS and ICMR, estimated with the three different models for each sex/cohort. We present the mean risk and the distribution of the individuals in the three risk categories stated above (0 –10%, 10 – 30%, and > 30%).

### Effect of the recalibrated prognostic model on treatment

**Figure 2.**
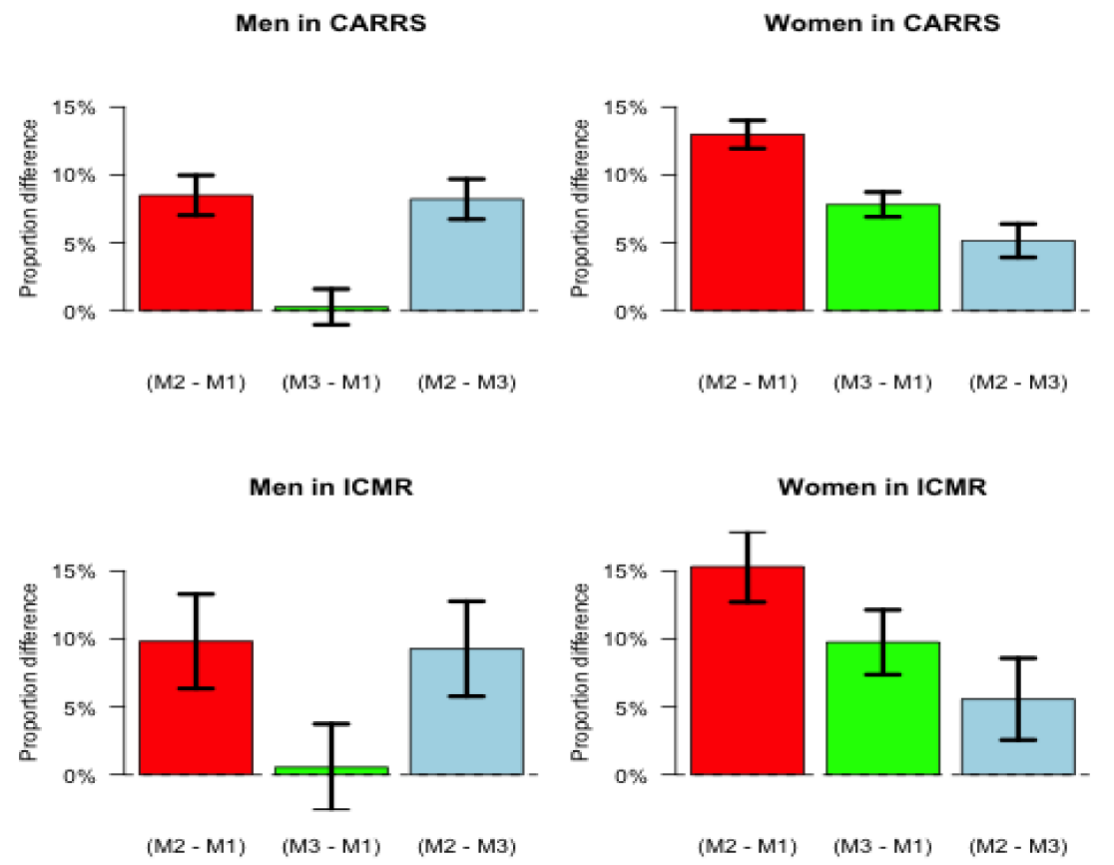
Difference in the proportion of treated individuals (risk > 30%) between the estimation of the three methods (M2-M1, M3-M1, and M2-M3). Bars reach the difference in proportions and segments represent 95% confidence intervals for the difference. M-1) Framingham M-2) F-CARRS recalibrated and M-3) F- ICMR recalibrated. F: Framingham; CARRS: Centre for cArdiometabolic Risk Reduction in South-Asia; ICMR: Indian Council of Medical Research

According to the WHO guidelines individuals with a risk of fatal or non-fatal cardiovascular event > 30% should get treatment with statins ^23^. In Figure 2 below we plot the difference in the proportion of individuals (by sex and cohort) that should be eligible for treatment according to the different models. For example, as shown in the red bar to the top-left graph of Figure 2, if we used model M2 instead of M1, about 8% more men in CARRS will be eligible for treatment. This can be calculated from the difference of 21.0% – 13.3% in Table 5 (first two rows for men). The pattern is very similar in both datasets within each sex. In men model M2 increases the proportion of men that should be treated compared to both model M1 and model M3, and M1 and M3 categorize men very similarly. For women, models M2 and M3 also increase the proportion of women that should be treated in comparison to M1, but in addition model, M2 also categorize more women than M3 as eligible to be treated.

**Table 5.** Means of estimated risks and distribution by risk categories of individuals in each cohort with different estimation models: M-1) Framingham M-2) F-CARRS recalibrated and M-3) F- ICMR recalibrated. F: Framingham; CARRS: Centre for cArdiometabolic Risk Reduction in South-Asia; ICMR: Indian Council of Medical Research.

	MEN				WOMEN			
Data/model	Mean	<=10%	10–30%	>30%	Mean	<=10%	10–30%	>30%
**CARRS participants:**								
M1) Framingham	0.131	58.7%	29.2%	**12.1%**	0.060	82.0%	14.9%	**3.1%**
M2) F-CARRS	0.182	47.2%	32.2%	**20.6%**	0.149	60.2%	23.6%	**16.1%**
M3) F-ICMR	0.133	58.2%	29.4%	**12.4%**	0.114	67.6%	21.5%	**11.0%**
**ICMR participants:**								
M1) Framingham	0.155	53.4%	30.7%	**16.0%**	0.079	76.5%	17.9%	**5.6%**
M2) F-CARRS	0.213	41.6%	32.6%	**25.8%**	0.188	51.7%	27.4%	**20.9%**
M3) F-ICMR	0.157	53.0%	30.5%	**16.5%**	0.145	60.6%	24.1%	**15.3%**

## Discussion

In this study, we calculated the 10-year Framingham CVD score in two cohorts of Indian urban populations (CARRS and ICMR) by using coefficients from a simplified Framingham model. We then predicted the risk in each cohort using three different models: one with the original Framingham reference coefficients and two recalibrated with the average risk factors prevalence in each of the datasets and the CVD mortality estimations for India from WHO-2012 data.

The average 10-year CVD risk estimates calculated using the Framingham recalibrated equation with the CARRS data was substantially higher than the original Framingham equation for both men and women, but the recalibrated equation using the ICMR the averages were only distinctly higher for women but not for men. A previous study in rural India, also found that the Framingham score underestimates in comparison with the one recalibrated with national data ^16^. Other studies in South Asian Indian populations have also shown higher CVD incidence in comparison to the predicted by Framingham risk score ^24– 27^.

The overall CVD risk score is used to inform clinical decisions to start treatment to lower blood pressure and statins. The thresholds recommended vary according to the guideline and settings. For example, the WHO guidelines recommend that individuals with a 30% 10-year risk of a fatal or non-fatal cardiovascular event should start with statins. Our study exemplifies that by using the prognostic model recalibrated with the CARRS data, there will be a substantial increase in the proportion of men and women that would be eligible for treatment with statins in comparison to the original Framingham risk score. However, by using the prognostic model recalibrated with the ICMR data, there would only be a substantial increase in the proportion of women that would be eligible for treatment.

To the best of the authors’ knowledge, this is the first large community-based study to recalibrate Framingham risk score in an urban population in India. One of our strengths is that the data are representative of their respective cities and that we used two different cohort studies. The main limitation is that we cannot check if the re-classification of the recalibrated model is indeed an improvement in risk prediction comparison with the original Framingham score because of the lack of cardiovascular events in the existing cohorts. Also, for the recalibration we used WHO mortality data which includes a population with and without previous CVD, which might have overestimated the risk, however, until large and robust cohorts ,with detailed outcome assessment, become available this is the best available dataset that can be used to estimate the expected mortality in patients without CVD.

Early identification and initiation of intensive primary prevention among individuals with high risk of CVDs are critically important in reducing the CVD burden in India. Although, almost all the major recent international guidelines including the National Institute for Health and Care Excellence (NICE) 2014 guidelines, World Health Organization (WHO) 2007 guidelines, European Society of Cardiology (ESC) 2016 guidelines and the 2017 American College of Cardiology (ACC)/American Heart Association (AHA) guidelines and national guideline unanimously recommend assessment of cardiovascular risk ^18, 28– 31^, their adoption in primary prevention is suboptimal ^31– 33^. Few of common barriers for its decreased use are; lack of national guidelines, too many choices for CVD risk score, the uncertainty of validity of these risk score model in local context, time-consuming and lack of adjustment for the treatment ^34, 35^. Recalibrated model based on local population can improve the validity of the risk score model and reduce the perceived barriers of physician related to the local validity and enhance the use of CVD prediction model in the clinical setting for primary prevention. However, our study shows that even recalibrated models using data from the same country could be indeed very different and therefore it is vital to recalibrate models applying relevant local data (reflecting as best as possible local prevalence and overall mortality data). With the increasing use of technology, a possible approach could be to develop risk calculators in which local prevalent data and local incidence data is easily uploaded, and a “tailored” recalibrated model is provided for each setting. However, in the long-term future studies should develop CVD prognostic models using high quality and well powered local cohorts (with outcome data) and evaluate their implementation and impact.

Valid and reliable local prognostic models will also be key to evaluate different high risk prevention strategies.

## Ethics and consent

CARRS (Centre for cArdiometabolic Risk Reduction in South-Asia) study was approved by the Institutional Review Boards (IRBs) of the Public Health Foundation of India, New Delhi (approval number: IRB00006658), All India Institute of Medical Sciences, New Delhi, Madras Diabetes Research Foundation, Chennai, India, Aga Khan University, Karachi, Pakistan, and Emory University, Atlanta, USA. ICMR (Indian Council of Medical Research) study was approved by All India Institute of Medical Sciences, New Delhi, ethics approval number is IEC/OP-05/06/09/10.

## Data availability

### Underlying data

This paper uses datasets from other studies. We gained access to these datasets by requesting permission from the PIs of the concerned studies. Therefore, data sets cannot be shared publicly.

Researchers who are interested can take permission from Dr. Prabhakaran who is the PI for both the studies (email id: dprabhakaran@ccdcindia.org) to access the data.

Extended data: We have added two supplementary figures:

- S1 Figure: This figure demonstrates contribution of risk factors to mean score. https://doi.org/10.6084/m9.figshare.11282900.v1
- S2 Figure: This figure shows predicted risk according to recalibration. https://doi.org/10.6084/m9.figshare.11282960.v1

### Reporting guidelines

Open Science Framework: TRIPOD statement ‘Cardiovascular risk prediction in India: Comparison of the original and recalibrated Framingham prognostic models in urban populations’. https://dx.doi.org/10.17605/OSF.IO/NXMZQ ^21^

Data are available under the terms of the Creative Commons Zero “No rights reserved” data waiver (CC0 1.0 Public domain dedication).
