# Supplementary material for: Cardiovascular risk prediction in India: Comparison of the original and recalibrated Framingham prognostic models in urban populations
Source: Wellcome Open Res. 2019 Dec 2;4:71. Originally published 2019 Apr 17. [Version 2] doi: 10.12688/wellcomeopenres.15137.2 (PMC7255911; doi:10.12688/wellcomeopenres.15137.2)
Supplement: Peer review history

# Peer review: Reviewer response for version 2

**DOI:** 10.21956/wellcomeopenres.17104.r37239

**Published:** 2020-05-26

**Authors:** Kay Tee Khaw

**Affiliations:** 1 Gonville and Caius College, University of Cambridge, Cambridge, UK

The authors have addressed and clarified the points raised in the review, in particular, the distinction between cardiovascular disease or coronary heart disease in the risk prediction algorithms, and provided explanations for the somewhat unexpected sex differences. Their main conclusion remains important and highly relevant, which is the need to have relevant local population data when making clinical or public health policy.

Is the work clearly and accurately presented and does it cite the current literature?

Yes

If applicable, is the statistical analysis and its interpretation appropriate?

Partly

Are all the source data underlying the results available to ensure full reproducibility?

No

Is the study design appropriate and is the work technically sound?

Yes

Are the conclusions drawn adequately supported by the results?

Yes

Are sufficient details of methods and analysis provided to allow replication by others?

Yes

Reviewer Expertise:

Epidemiologist.

I confirm that I have read this submission and believe that I have an appropriate level of expertise to confirm that it is of an acceptable scientific standard.

# Peer review: Reviewer response for version 2

**DOI:** 10.21956/wellcomeopenres.17104.r37238

**Published:** 2019-12-16

**Authors:** Katrina K. Poppe

**Affiliations:** 1 Department of Medicine, University of Auckland, Auckland, New Zealand

Thank you for considering my comment about people in the cohorts with CVD.

Is the work clearly and accurately presented and does it cite the current literature?

Partly

If applicable, is the statistical analysis and its interpretation appropriate?

Yes

Are all the source data underlying the results available to ensure full reproducibility?

Partly

Is the study design appropriate and is the work technically sound?

Yes

Are the conclusions drawn adequately supported by the results?

Yes

Are sufficient details of methods and analysis provided to allow replication by others?

Yes

Reviewer Expertise:

NA

I confirm that I have read this submission and believe that I have an appropriate level of expertise to confirm that it is of an acceptable scientific standard.

# Peer review: Reviewer response for version 1

**DOI:** 10.21956/wellcomeopenres.16516.r36285

**Published:** 2019-09-11

**Authors:** Katrina K. Poppe

**Affiliations:** 1 Department of Medicine, University of Auckland, Auckland, New Zealand

Overall

 This paper has a clear and valid rationale, and clearly describes the approach used to recalibrate the available risk score to the two Indian cohorts. The discussion is clear and I agree with the main conclusion that “it is essential to develop high quality and well powered local cohorts (with outcome data) to develop local prognostic models”.

Main comments

 The authors note that there is “no validated prognostic model for an Indian urban population” and so focus on recalibrating a Framingham equation developed in a completely different population. In 2018, the VIEW/PREDICT group in New Zealand published sex-specific risk scores for the primary prevention of CVD (Pylypchuk *et al.* Lancet 2018) ^1^. As the scores were derived from a multi-ethnic cohort, they included a categorical variable for ethnicity, allowing risk to vary for the five largest defined ethnic groups in the country, one of which was Indian (derived from 14,188 Indian women and 20,232 Indian men). Although the meaning of urban and rural will differ by country, the PREDICT cohort will be predominantly urban. I would suggest that the PREDICT risk scores are a more appropriate starting point for assessing risk in an Indian population.

 The CARRS and ICMR cohorts are population studies derived from sampling individuals per household, thus each cohort will include a mixture of people with and without CVD. I could not see anywhere that people with CVD were excluded, so my following comments assume they have remained in the cohorts.

- The Framingham equation being referred to in this study is for people without CVD, and from the risk factors included, seems to be the 2008 score by D’Agostino *et al*. ^2^ (the equation is not referenced in the current manuscript). What proportion of each cohort already had CVD and what was the composition of the disease? E.g., proportions with stroke vs MI vs peripheral vascular disease vs… etc. Could a difference in that composition have led to the difference in risk estimates using recalibrated risk scores in the two cohorts?
- The reference survival (So) was based on WHO estimated country-level mortality rates. This would be all-cause mortality, yet I believe the fatal component of the outcome predicted in the 2008 Framingham score is “coronary death”. It is fair enough to then estimate the non-fatal events from a ratio, but it will multiplicatively amplify the non-fatal CVD event rate if based on all-cause deaths.
- The country-level mortality from WHO will include people from rural and urban areas, and people with and without existing CVD. Either of these factors may reduce its relevance to the cohorts being studied, and/or the risk score being investigated. 

A wording suggestion - the “Framingham score calculation (ie. Xi)” introduced on page 3 is more standardly called the prognostic index or linear predictor.

 As the authors note, a key limitation of the available data is that the predicted risks cannot be compared to actual event rates in the cohorts, so there is no way of knowing which version of the score is accurate, and if recalibration is even needed. This further supports the need for development of high quality local cohorts with outcome data.

Is the work clearly and accurately presented and does it cite the current literature?

Partly

If applicable, is the statistical analysis and its interpretation appropriate?

Yes

Are all the source data underlying the results available to ensure full reproducibility?

Partly

Is the study design appropriate and is the work technically sound?

Yes

Are the conclusions drawn adequately supported by the results?

Yes

Are sufficient details of methods and analysis provided to allow replication by others?

Yes

Reviewer Expertise:

NA

I confirm that I have read this submission and believe that I have an appropriate level of expertise to confirm that it is of an acceptable scientific standard, however I have significant reservations, as outlined above.

## References

[1] : Cardiovascular disease risk prediction equations in 400 000 primary care patients in New Zealand: a derivation and validation study. *The Lancet*.2018;391(10133) : 10.1016/S0140-6736(18)30664-0 1897-1907 10.1016/S0140-6736(18)30664-0 29735391

[2] : General cardiovascular risk profile for use in primary care: the Framingham Heart Study. *Circulation*.2008;117(6) : 10.1161/CIRCULATIONAHA.107.699579 743-53 10.1161/CIRCULATIONAHA.107.699579 18212285

# Response

**Published:** 2019-11-25

**Authors:** Priti Gupta

**Affiliations:** Center for Chronic Disease Control, India

We thank the opportunity to respond to the reviewer very positive and helpful comments, which we have now responded point-by-point and, by doing so, we believe it has improved our manuscript

Overall

This paper has a clear and valid rationale, and clearly describes the approach used to recalibrate the available risk score to the two Indian cohorts. The discussion is clear and I agree with the main conclusion that “it is essential to develop high quality and well powered local cohorts (with outcome data) to develop local prognostic models”.

Main comments

The authors note that there is “no validated prognostic model for an Indian urban population” and so focus on recalibrating a Framingham equation developed in a completely different population. In 2018, the VIEW/PREDICT group in New Zealand published sex-specific risk scores for the primary prevention of CVD (Pylypchuk *et al.* Lancet 2018) ^1^. As the scores were derived from a multi-ethnic cohort, they included a categorical variable for ethnicity, allowing risk to vary for the five largest defined ethnic groups in the country, one of which was Indian (derived from 14,188 Indian women and 20,232 Indian men). Although the meaning of urban and rural will differ by country, the PREDICT cohort will be predominantly urban. I would suggest that the PREDICT risk scores are a more appropriate starting point for assessing risk in an Indian population.

The CARRS and ICMR cohorts are population studies derived from sampling individuals per household, thus each cohort will include a mixture of people with and without CVD. I could not see anywhere that people with CVD were excluded, so my following comments assume they have remained in the cohorts.

- The Framingham equation being referred to in this study is for people without CVD, and from the risk factors included, seems to be the 2008 score by D’Agostino *et al*. ^2^ (the equation is not referenced in the current manuscript). What proportion of each cohort already had CVD and what was the composition of the disease? E.g., proportions with stroke vs MI vs peripheral vascular disease vs… etc. Could a difference in that composition have led to the difference in risk estimates using recalibrated risk scores in the two cohorts?
- The reference survival (So) was based on WHO estimated country-level mortality rates. This would be all-cause mortality, yet I believe the fatal component of the outcome predicted in the 2008 Framingham score is “coronary death”. It is fair enough to then estimate the non-fatal events from a ratio, but it will multiplicatively amplify the non-fatal CVD event rate if based on all-cause deaths.
- The country-level mortality from WHO will include people from rural and urban areas, and people with and without existing CVD. Either of these factors may reduce its relevance to the cohorts being studied, and/or the risk score being investigated. 

**We thank for the reviewer for these thoughtful comments. We can confirm that the original analyses excluded patients with previous CVD in the ICMR cohort, but we have not excluded them in the CARRS cohort. Therefore, following the suggestion from the reviewer we have re-done the analysis (results remain very similar) and had made this clear in the methods. We have also updated all tables and figures accordingly. We agree that WHO data will include data with and without existing CVD but this is the best dataset that it is available to estimate the CVD mortality in India. While large and robust cohorts are unavailable re-calibration of existing models using the existing local data is the best approach we can take. We have added this as a potential limitation in the discussion.**

A wording suggestion - the “Framingham score calculation (ie. Xi)” introduced on page 3 is more standardly called the prognostic index or linear predictor.

**We have updated the text following the reviewer’s suggestion on page 3**

# Peer review: Reviewer response for version 1

**DOI:** 10.21956/wellcomeopenres.16516.r35349

**Published:** 2019-04-30

**Authors:** Kay Tee Khaw

**Affiliations:** 1 Gonville and Caius College, University of Cambridge, Cambridge, UK

**General comments**

 Estimating an individual’s absolute risk of cardiovascular disease within the next 5 or 10 years is the basis of the “individual high risk” strategy to prevention of cardiovascular disease; those individuals identified at highest risk are targeted for established preventive interventions such as medications to lower blood cholesterol or blood pressure levels to reduce cardiovascular disease risk.

 Various risk algorithms are used, most of them based on some variant of the original Framingham cardiovascular risk score, using classical cardiovascular risk factors blood cholesterol, blood pressure, smoking, body mass index and diabetes as well as age and sex.

 The accuracy of risk algorithms for predicting cardiovascular disease risk in an individual therefore depends on a number of factors:

 Firstly, we require coefficients of risk for the different risk factors, that is how much the level or presence of a risk factor may increase future cardiovascular disease risk. While most may use a standard coefficient for each risk factor such as blood pressure, or smoking there may be variants on this such as different coefficients for different age groups, or men and women.

 Secondly we require the incidence of cardiovascular disease over the next ten years; again, these will differ in men and women and in different age groups as well as different populations, and indeed over time with secular trends.

 In addition, in terms of public health implications, the proportion or numbers in the population who might be classified as high risk will also depend on the prevalence of the relevant risk factors in the different groups.

 The risk algorithms generally used in clinical and public health, such as the Framingham cardiovascular disease risk score are largely derived from data based originally on the Framingham Study or more recently variants of algorithms from other prospective studies mostly based in Western countries.

 The authors of this study make the very strong argument that though the burden of cardiovascular disease in India is huge, and increasing, there is a dearth of data on the use of such risk algorithms as applied to the Indian population, and no validated prognostic model for an Indian urban population

 They make the laudable attempt to recalibrate the original Framingham model using data from two urban Indian studies. They estimated three risk score equations using three different models. The first model was based on the Framingham original model, the second and third were recalibrated models using risk factor prevalence from the CARRS and ICMR studies and estimated survival from WHO 2012 data for India. They applied the three risk scores to the CARRS and ICMR participants and estimated the proportions of those at high risk (>30% 10 year CVD risk). The estimates of the proportions with such risk varied greatly using the different models.

 Their main conclusion was that their study exemplifies the variation between recalibrated models using different data from the same country. They state that it is essential to develop high quality and well powered local cohorts with outcome data to develop local prognostic models. While I would agree with the overall conclusions of the authors and support strongly the need for locally relevant population studies, there are a number of points that should be clarified in the text.

**Issues to consider in the manuscript**

 The authors in this exercise state that they have recalibrated the original Framingham model using data from two urban Indian studies. They estimated three risk score equations using the first model based on the Framingham original model, the second and third were recalibrated models using risk factor prevalence from two Indian studies, the CARRS and ICMR studies and estimated survival from WHO 2012 data for India.

 A general comment: the text mostly refers to CVD (cardiovascular disease) risk but sometimes refers to CHD (coronary heart disease) risk. These are not the same: CVD includes CHD but is a larger category encompassing other conditions such as stroke etc so absolute rates are higher, and the relationship to risk factors is somewhat different. On page 3, there is a statement that the Framingham risk equation is a... method to measure coronary heart disease (CHD) risk but then later under Step 1 the Framingham score it is stated that this is it was calculated for CVD risk. Then in Step 2 the authors state that they obtained 2012 yearly mortality rates for CHD from India. Were the estimates for CVD or CHD? This materially affects the estimates of proportion with any given 10 year absolute risk. In particular, there is a large male:female excess for coronary heart disease but generally stroke rates are more similar between men and women. This needs to be clarified throughout the manuscript.

 Most approaches estimating the proportion of those with a given 10 year CVD (or CHD) risk in a defined population (e.g. >30% 10 years or >20% 10 years) generally apply the Framingham coefficients to derive individual Framingham risk scores. The proportion with a high score thus relies both on the coefficients used and the prevalence of risk factors in the population which may vary in different populations. This can be done with cross sectional data in which data on risk factors are available using the Framingham risk score. This was done in Step 1 by the authors using cross sectional data from the two Indian studies: CARRS and ICMR.

 The authors then proceeded to develop a model based on 2012 yearly mortality rates for CHD (as stated in the text rather than CVD) for India from WHO (page 3, Step 2). From what I understand, they then derived the average probability of not having events in 10 years for men and women separately. It would seem that because a longitudinal population cohort with individual follow up and endpoints was not available, the WHO data were used to approximate the data to estimate the score of the “average individual in the population by multiplying the averages of the variables by the original Framingham coefficients and adding the values for all risk factors”.

 Then in Step 4 they calculated the risks for everyone in the CARRS and ICMR data sets with the 3 models using different combinations of reference score and survival probabilities.

 I found Steps 2-4 rather hard to follow and it would particularly have been helpful to know more about the assumptions they used in applying the WHO 2012 mortality dataset (apart from the CHD/CVD distinction). For example, they state they used the total CHD annual mortality- was this age standardised, or if not, was this for the whole population or a subset of the population? Presumably the CARRS and ICMR studies encompassed the whole adult age range but it would be helpful to have some more information about the age distribution the rates of cardiovascular disease are so strongly age related. The estimates of the proportion of the population at high cardiovascular risk over 10 years must surely depend hugely on the age distribution of the population, and the Framingham algorithms were derived on a slightly older age distribution than 20year + (CARRS).

 Table 1 shows coefficients from the simplified Framingham model. There are a number of references to the Framingham model cited which I have looked up, and I may have missed this, but it would be helpful to cite the exact reference for coefficients used in this table. I was rather surprised for example, in the sex specific coefficients that the women had different coefficients from men for many of the risk factors, whereas in many of the Framingham algorithms that I have seen, the sexes are combined for risk factors with a weighting for male vs female. Body mass index, age, and smoking appear to be more weighted in men whereas blood pressure, and diabetes are more highly weighted in women. This is relevant when considering results.

 The tables are clearly presented showing the risk factor distribution in the two population datasets from India (Table 2) and reference scores and survivals in Table 3. The examples of reference score calculations using means from the CARRS population in Table 4 is also of interest with similar differential weighting of the risk factors in men and women as with the Framingham study.

 However, the results that were surprising were that women in Tables 4 and Figure 1 appeared to have higher Framingham scores. In Table 5, however, it seems that while there was a much higher proportion of men with >30% risk compared to women using the Framingham risk score, the proportions of men and women were much more similar using the F-CARRS or F-ICMR derived scores. Given that the prevalence of risk factors in women in both CARRS and ICMR were generally lower in women than men apart from body mass index (Table 1), and that women had lower mortality (CHD or CVD) than men this does not really make sense. I wonder whether this may be a consequence of the modelling, (CHD/CVD mortality) or insufficient account of the age distribution. Perhaps they could check their models.

**Minor points**

 The introduction makes a strong argument about the importance of cardiovascular disease in India. However, some of the statements may need some nuance. The statement that the all age death rate increased significantly between 1990 and 2016 for ischemic heart disease. This may reflect both increasing age specific mortality from heart disease or major demographic shifts that is ageing of the population as ischemic heart disease rates increase with age and the numbers and proportion of older people in India have increased over that time period. (In the USA, absolute CVD deaths have increased over the last few decades despite declining age specific or age standardised rates, simply because of the increased numbers of older people). Similarly though, the statement that Indians are affected by CVDs at a younger age compared to their European counterparts may well be true, the statement that more than 50% CVD deaths occur before the age of 70 years may reflect the much younger age distribution of the population – comparisons of risk are not robust without appropriate denominators.

 Though the methods used and estimates of proportions at different levels of absolute risk can be discussed, the authors have made the point clearly. I think this manuscript indeed does illustrate and highlight the extraordinary discrepancy between the large and increasing burden of cardiovascular disease in emerging economies globally and the paucity of locally relevant data; they have provided an example of where such local data can be used and the great need for relevant evidence and support of ongoing population studies to inform policy and practice.

 As an aside, though not the focus of this manuscript, whatever the model used, the estimates indicate the very high proportion of the population with high absolute cardiovascular risk and challenges of the individual high risk strategy in countries such as India. The discussion is careful and considered in terms of the various guidelines for identifying high risk individuals for preventive interventions but perhaps might also point out the value of local population studies for providing the evidence base for mass preventive strategies.

Is the work clearly and accurately presented and does it cite the current literature?

Yes

If applicable, is the statistical analysis and its interpretation appropriate?

Partly

Are all the source data underlying the results available to ensure full reproducibility?

No

Is the study design appropriate and is the work technically sound?

Yes

Are the conclusions drawn adequately supported by the results?

Yes

Are sufficient details of methods and analysis provided to allow replication by others?

Yes

Reviewer Expertise:

Epidemiologist.

I confirm that I have read this submission and believe that I have an appropriate level of expertise to confirm that it is of an acceptable scientific standard, however I have significant reservations, as outlined above.

# Response

**Published:** 2019-11-25

**Authors:** Priti Gupta

**Affiliations:** Center for Chronic Disease Control, India

We thank the opportunity to respond to the reviewers’ very positive and helpful comments, which we have now responded point-by-point and, by doing so, we believe it has improved our manuscript

REVIEWER 1

- A general comment: the text mostly refers to CVD (cardiovascular disease) risk but sometimes refers to CHD (coronary heart disease) risk. These are not the same: CVD includes CHD but is a larger category encompassing other conditions such as stroke etc so absolute rates are higher, and the relationship to risk factors is somewhat different. On page 3, there is a statement that the Framingham risk equation is a... method to measure coronary heart disease (CHD) risk but then later under Step 1 the Framingham score it is stated that this is it was calculated for CVD risk. Then in Step 2, the authors state that they obtained 2012 yearly mortality rates for CHD from India. Were the estimates for CVD or CHD? This materially affects the estimates of proportion with any given 10-year absolute risk. In particular, there is a large male: female excess for coronary heart disease but generally stroke rates are more similar between men and women. This needs to be clarified throughout the manuscript.

**Response: We thank the reviewer for identifying this inconsistency. The Framingham score we used estimates the probability of CVD and that is what we meant and accordingly we have obtained the corresponding India CVD 2012 mortality rate. We have now edited the manuscript accordingly. **

- Most approaches estimating the proportion of those with a given 10 year CVD (or CHD) risk in a defined population (e.g. >30% 10 years or >20% 10 years) generally apply the Framingham coefficients to derive individual Framingham risk scores. The proportion with a high score thus relies both on the coefficients used and the prevalence of risk factors in the population which may vary in different populations. This can be done with cross sectional data in which data on risk factors are available using the Framingham risk score. This was done in Step 1 by the authors using cross sectional data from the two Indian studies: CARRS and ICMR.
  The authors then proceeded to develop a model based on 2012 yearly mortality rates for CHD (as stated in the text rather than CVD) for India from WHO (page 3, Step 2). From what I understand, they then derived the average probability of not having events in 10 years for men and women separately. It would seem that because a longitudinal population cohort with individual follow up and endpoints was not available, the WHO data were used to approximate the data to estimate the score of the “average individual in the population by multiplying the averages of the variables by the original Framingham coefficients and adding the values for all risk factors”. Then in Step 4 they calculated the risks for everyone in the CARRS and ICMR data sets with the 3 models using different combinations of reference score and survival probabilities. I found Steps 2-4 rather hard to follow and it would particularly have been helpful to know more about the assumptions they used in applying the WHO 2012 mortality dataset (apart from the CHD/CVD distinction). For example, they state they used the total CHD annual mortality- was this age standardised, or if not, was this for the whole population or a subset of the population? Presumably the CARRS and ICMR studies encompassed the whole adult age range but it would be helpful to have some more information about the age distribution the rates of cardiovascular disease are so strongly age related. The estimates of the proportion of the population at high cardiovascular risk over 10 years must surely depend hugely on the age distribution of the population, and the Framingham algorithms were derived on a slightly older age distribution than 20year + (CARRS).

 

**Response: We thank the reviewer for the comments, and we hope the answer below clarified the procedure we followed**

1. **We did not develop a “ *de novo*” risk score model (i.e. we cannot calculate coefficients for the different risk factors because, as the reviewer points out, we have no follow up and outcomes), we use instead the coefficients from the existing Framingham model. What we did is to re-calibrate this model to the Indian data that we are going to predict.**
2. **To re-calibrate a model in a new dataset we need a “reference individual”, i.e. an individual with a known value of Framingham score and known risk of having a cardiovascular event.The risk of any other individual is calculated depending on the difference between that individual’s score and the reference individual’s score.The question is then, how to find the reference individual. In reality no one has a reference individual in our data, so we have to make assumptions. One option is to assume that the population CV risk estimated by the WHO studies would be the risk of a person with average score in the Framingham model in our Indian sample. And so the reference individual will be a person with score produced by setting all variables to the average value and we will assume that their risk is precisely the population CVD risk estimated by WHO. **
3. **WHO estimates of death rates were calculated standardizing by age with the WHO standard population for individuals between 30 and 70 years old.**

- Table 1 shows coefficients from the simplified Framingham model. There are a number of references to the Framingham model cited which I have looked up, and I may have missed this, but it would be helpful to cite the exact reference for coefficients used in this table. I was rather surprised for example, in the sex specific coefficients that the women had different coefficients from men for many of the risk factors, whereas in many of the Framingham algorithms that I have seen, the sexes are combined for risk factors with a weighting for male vs female. Body mass index, age, and smoking appear to be more weighted in men whereas blood pressure, and diabetes are more highly weighted in women. This is relevant when considering results.

**Response: We have indeed used the Framingham sex specific coefficients available on supplementary table 1 of D’Agostino *et al*. General Cardiovascular Risk Profile for Use in Primary Care The Framingham Heart Study. Circulation. 2008;117: 743-753. We have now made it clearer and included this reference in the methods and tables i.e new reference number 3.**

- The tables are clearly presented showing the risk factor distribution in the two population datasets from India (Table 2) and reference scores and survivals in Table 3. The examples of reference score calculations using means from the CARRS population in Table 4 is also of interest with similar differential weighting of the risk factors in men and women as with the Framingham study. However, the results that were surprising were that women in Tables 4 and Figure 1 appeared to have higher Framingham scores. In Table 5, however, it seems that while there was a much higher proportion of men with >30% risk compared to women using the Framingham risk score, the proportions of men and women were much more similar using the F-CARRS or F-ICMR derived scores. Given that the prevalence of risk factors in women in both CARRS and ICMR were generally lower in women than men apart from body mass index (Table 1), and that women had lower mortality (CHD or CVD) than men this does not really make sense. I wonder whether this may be a consequence of the modelling, (CHD/CVD mortality) or insufficient account of the age distribution. Perhaps they could check their models.

**Response: We thank the reviewer for this comment, and we understand the results appear to be counter-intuitive if one considers that women seem to have lower levels of risk factor than men in this population. However, it is important to consider the coefficients multiplying the risk factors when we calculate the score. For example, the coefficients for SBP, both treated and untreated, are higher in women than men, and this is a key factor that adds much weight to the score. Even if women have lower SBP mean than men, when multiplied by the coefficient this increases more their score than in men. To illustrate the contribution of each risk factor we have added a figure in the appendix (see Figure-S1).  It can be seen that age and BMI actually contribute to increase the average score of men more than for women, but SBP (both treated and untreated) contribute more to increase the score in women. Adding up all these contributions women end up with a higher mean score than men in both data sets.**

**Finally, the conversion of the score to a risk factor depends on the re-calibration (the choice of “reference individual” as explained in our response to your question 2).   If the score of the reference individual is lowered but while their risk is kept the same, the predicted risks of all other individuals in the data will increase.  To illustrate this, we have created another plot (see Figure-S2) where we show how the same distribution of risk scores is converted into different distribution of predicted risks depending on the recalibration model.**

Minor points

- The introduction makes a strong argument about the importance of cardiovascular disease in India. However, some of the statements may need some nuance. The statement that the all age death rate increased significantly between 1990 and 2016 for ischemic heart disease. This may reflect both increasing age specific mortality from heart disease or major demographic shifts that is ageing of the population as ischemic heart disease rates increase with age and the numbers and proportion of older people in India have increased over that time period. (In the USA, absolute CVD deaths have increased over the last few decades despite declining age specific or age standardised rates, simply because of the increased numbers of older people). Similarly though, the statement that Indians are affected by CVDs at a younger age compared to their European counterparts may well be true, the statement that more than 50% CVD deaths occur before the age of 70 years may reflect the much younger age distribution of the population – comparisons of risk are not robust without appropriate denominators.

**Response: We agree with the reviewer that there are two issues regarding the statement that CVD affect Indian as a younger age. We have now made it clear that it related both to a demographic age distribution of age plus an increased in age standardized age for CVD.**

**We have added following line with reference:**

**“Age adjusted prevalence of CVDs have also increased in India(1,2)”**

- Though the methods used and estimates of proportions at different levels of absolute risk can be discussed, the authors have made the point clearly. I think this manuscript indeed does illustrate and highlight the extraordinary discrepancy between the large and increasing burden of cardiovascular disease in emerging economies globally and the paucity of locally relevant data; they have provided an example of where such local data can be used and the great need for relevant evidence and support of ongoing population studies to inform policy and practice. As an aside, though not the focus of this manuscript, whatever the model used, the estimates indicate the very high proportion of the population with high absolute cardiovascular risk and challenges of the individual high risk strategy in countries such as India. The discussion is careful and considered in terms of the various guidelines for identifying high risk individuals for preventive interventions but perhaps might also point out the value of local population studies for providing the evidence base for mass preventive strategies.

**Response: We thank the reviewer for the positive comments about our discussion and we agree about the implications of local studies for providing evidence for preventive strategies. We have now edited the discussion section accordingly and inlcuded following lines:**

**“Also, for the recalibration we used WHO mortality data which includes a population with and without previous CVD, which might have overestimated the risk, however, until large and robust cohorts ,with detailed outcome assessment, become available this is the best available dataset that can be used to estimate the expected mortality in patients without CVD.”**

**“Valid and reliable local prognostic models will also be key to evaluate different high risk prevention strategies.”**
